# Man With Blurry Vision

**DOI:** 10.1016/j.acepjo.2026.100514

**Published:** 2026-09-23

**Authors:** Ping-Ting Wu, Chee-Fah Chong

**Affiliations:** 1 Department of Emergency Medicine, Shin-Kong Wu Ho-Su Memorial Hospital, Taipei City, Taiwan; 2 School of Medicine, Fu Jen Catholic University College of Medicine, New Taipei City, Taiwan

## 1 Case Presentation

A 60-year-old male with no documented medical history presented to the emergency department with acute painless monocular visual loss in the right eye (light perception only). Intraocular pressure (IOP) measured 21.3 mm Hg, and a distinct horizontal layer of whitish material was noted at the bottom of the anterior chamber on slit-lamp examination (Fig. A). He was discharged with a diagnosis of mature cataract and phacolytic glaucoma without systemic workup.

**Figure:**
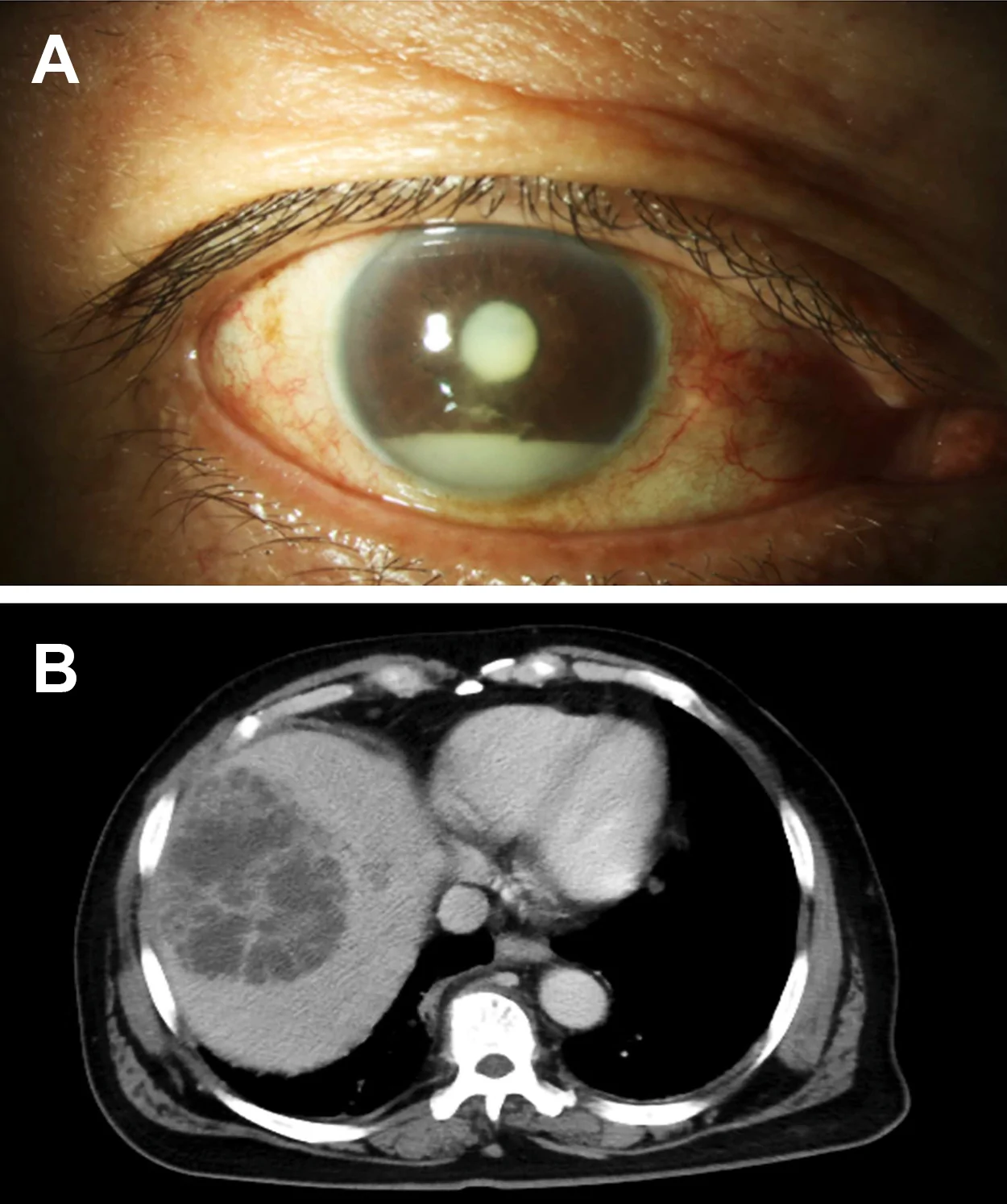
(A) Eye exam reveals a mature white cataract and a distinct horizontal layer of whitish material visible at the bottom of the anterior chamber, suspected to be pseudohypopyon. (B) Contrast-enhanced CT scan showing a peripherally enhancing and centrally hypoattenuating, fluid-filled lesion, compatible with liver abscess. CT, computed tomography.

He returned 40 hours later febrile (39.3° C) with severe eye pain and further visual deterioration. Emergent contrast-enhanced CT revealed a 10.2 cm hepatic abscess (Fig. B) with bilateral pulmonary septic emboli. Laboratory workup confirmed new-onset diabetes mellitus (HbA1c 6.8%).

## 2 Diagnosis: Endogenous Endophthalmitis Secondary to *Klebsiella pneumoniae*

The revised diagnosis was endogenous endophthalmitis (EE) secondary to *Klebsiella pneumoniae* invasive syndrome.^1^ Phacolytic glaucoma typically presents with IOP exceeding 30 mm Hg; the borderline IOP of 21.3 mm Hg should have redirected suspicion toward EE, which characteristically produces intraocular inflammation with normal or reduced IOP.

Hypopyon in the absence of elevated IOP demands urgent exclusion of EE before attributing findings to a lens-related etiology. Emergent vitreous tapping with intravitreal antibiotics, hepatic drainage, and systemic antimicrobials were initiated.

This case illustrates that EE may be the earliest manifestation of occult systemic infection and undiagnosed diabetes, making metabolic screening and cross-sectional abdominal imaging essential components of the workup.^2^

## Funding and Support

By *JACEP Open* policy, all authors are required to disclose any and all commercial, financial, and other relationships in any way related to the subject of this article as per ICMJE conflict of interest guidelines (see www.icmje.org). The authors have stated that no such relationships exist.

## Informed Consent

Written informed consent to take part in the study and to publish the article has been obtained from all participants or their legal representatives. The privacy rights of participants have been observed. It was signed and attached in a separate document.

## Disclaimer

This study was performed in compliance with relevant laws, regulatory frameworks, and guidelines where the research took place. This study was approved by the Shin-Kong Memorial Hospital Institutional Review Board. (Approval No. SKH-03889080). This research follows the CARE guidelines and the CARE checklist.

## Conflict of Interest

All authors have affirmed they have no conflicts of interest to declare.
